# Mapping the Flavor Contributing Traits on "Fengwei Melon" (*Cucumis melo* L.) Chromosomes Using Parent Resequencing and Super Bulked-Segregant Analysis

**DOI:** 10.1371/journal.pone.0148150

**Published:** 2016-02-03

**Authors:** Hong Zhang, Hongping Yi, Mingzhu Wu, Yongbin Zhang, Xuejin Zhang, Meihua Li, Guangzhi Wang

**Affiliations:** Hami Melon Research Center, Xinjiang Academy of Agricultural Science, Urumqi, Xinjiang, China; University of Leicester, UNITED KINGDOM

## Abstract

We used a next-generation high-throughput sequencing platform to resequence the Xinguowei and Shouxing melon cultivars, the parents of Fengwei melon. We found 84% of the reads (under a coverage rate of “13×”) placed on the reference genome DHL92. There were 2,550,000 single-nucleotide polymorphisms and 140,000 structural variations in the two genomes. We also identified 1,290 polymorphic genes between Xinguowei and Shouxing. We combined specific length amplified fragment sequencing (SLAF-seq) and bulked-segregant analysis (super-BSA) to analyze the two parents and the F_2_ extreme phenotypes. This combined method yielded 12,438,270 reads, 46,087 SLAF tags, and 4,480 polymorphic markers (average depth of 161.81×). There were six sweet trait-related regions containing 13 differential SLAF markers, and 23 sour trait-related regions containing 48 differential SLAF markers. We further fine-mapped the sweet trait to the genomic regions on chromosomes 6, 10, 11, and 12. Correspondingly, we mapped the sour trait-related genomic regions to chromosomes 2, 3, 4, 5, 9, and 12. Finally, we positioned nine of the 61 differential markers in the sweet and sour trait candidate regions on the parental genome. These markers corresponded to one sweet and eight sour trait-related genes. Our study provides a basis for marker-assisted breeding of desirable sweet and sour traits in Fengwei melons.

## Introduction

The melon (*Cucumis melo* L.) is an important horticultural crop, and has diverse phenotypes and genotypes that result in variations in fruit quality including sugar and acid content, aroma, and fruit shape [1]. The principal component of fruit quality in sweet melons is the content of sugar, primarily sucrose [2–4]. Details of the metabolic pathways involved in sucrose accumulation in melon fruit have been previously described [5]. The organic acid content in most melon fruit is very low (pH >6.0) [6]. However, some varieties accumulate high levels of acid (pH <5) [7–11], and some breeders of melons have combined high acidity and high sugar traits to develop dessert cultivars [12–14]. The hereditary characteristics of citric acid content and titratable acid have been studied in melon fruit [7]. A single major QTL for pH has been found to co-localize with major QTLs for citric and malic acids [8]. Furthermore, the linkage map position for pH genes has been reported and an SSR marker was identified as being tightly associated with pH [10]. A previous study showed in bred melons that the high sugar and low pH traits were inherited independently of each other, and that the acidity trait was particularly associated with citric acid levels [12]. However, the relationship between genomic variations and the sour trait phenotype has not been reported in melon fruit.

Fengwei melon is a hybrid obtained from the Shouxing and Xinguowei varieties, and has a unique sweet and sour taste that results from the combined traits of its parents. Shouxing [*Cucumis melo* L. ssp. *melo cover*. *ameri* (Pang) Greb] is a crossbred from multiple parents, while Xinguowei [*Cucumis melo* L. ssp. *melo convar*. *ameri* (Pang) Greb, a sour tasting Hami melon line bred using high-dose Co^60^ γ-ray irradiation] has a unique sweet and sour taste [13].

Over the past few years, various genetic and genomic resources for melon have become available. New genetic maps have been reported for numerous traits such as fruit size and shape, climacteric ripening, netting, color, various metabolites, total soluble solids, and pH [8,9,15,16]. Resequencing technology and bioinformatics tools have helped to unravel the genetic variations and diversity on a genome-wide scale [17]. Garcia-Mas et al. [18] sequenced the complete genome of the double haploid line, DHL92, and provided valuable information about this important cucurbit. Blanca et al. [19] resequenced the transcriptomes of 67 melon genotypes using a high-throughput SOLiD^™^ system. Bulked-segregant analysis (BSA) is used for fine-mapping of phenotypic mutants [20] and is substantially enhanced by whole genome sequencing [21]. The use of sequence-based markers such as restriction site-associated DNA (RAD) markers, helps to achieve high-density discovery and genotyping in large populations [22]. However, these technologies usually fail in the presence of repetitive genomic sequences that usually are not useful in mapping experiments. Sun et al. [23] developed an enhanced reduced representation library (RRL) sequencing method, known as specific length amplified fragment sequencing (SLAF-seq) technology, that combines locus-specific amplification and high-throughput sequencing. This technique avoids the selection of repetitive genomic sequences and is highly accurate, low-cost, and fast.

Therefore, the present study aimed to localize important genes related to the sweet and sour traits in Fengwei melon (the F1 hybrid of the Shouxing and Xinguowei varieties). We used parental resequencing and comparative genomics to compare whole genome variations and functional genes between the two parental lines, in order to provide references for the gene mapping of sweet and sour traits. We used a combination of SLAF-seq and BSA technology (super-BSA) to help identifying the genomic regions affecting these traits in pools of extreme phenotypes from the F_2_ population (Fengwei melon ⊗). Our specific objectives were: 1) to obtain sweetness and sourness candidate genes through bioinformatics mapping of candidate regions closely correlated with sweet and sour traits; and 2) to perform a combined analysis of genes in these regions and SLAF differential markers.

## Materials and Methods

### Plants

The Shouxing parental line [*Cucumis melo* L. ssp. *melo convar*. *ameri* (Pang.) Greb] is a crossbred from multiple parents and its fruit has a sweet taste. The other parental line used in this study, Xinguowei [designated guowei (sour taste Hami melon line bred from the 76–2 line using high-dose Co^60^ γ-ray irradiation [13]) × PI140471], is a sour-tasting variety. Fengwei melon is the F_1_ hybrid obtained using Shouxing as the female parent and Xinguowei as the male parent. Its fruit has a unique sour and sweet taste. We obtained the F_2_ segregating population by self-pollinating the F_1_ hybrid (Fengwei melon).

Ten plants of each of the female parent, male parent, and F_1_ and F_2_ populations were grown at the experimental field of Xinjiang Academy of Agricultural Science (Xinjiang Turpan, China) in an open field during spring 2012 (F_2_ individuals, *n* = 479) and autumn 2012 (F_2_ individuals, *n* = 500), and in a greenhouse in Xinjiang Turpan during autumn 2012 (F_2_ individuals, *n* = 514). No endangered or protected species were involved in this study.

### Total soluble solids and pH of the fruit

A digital refractometer (Atago Co, Ltd, Tokyo, Japan) was used to measure the total soluble solids (TSS). A digital pH meter (Ohaus, Shanghai, China) was used to measure the pH of the juice [5]. The juice used in these experiments was extracted from a single fruit harvested from each plant. About 5 g of ripe flesh was taken from the equatorial portion of the fruit after removing the rind and seed cavity. The flesh was squeezed by hand, and the juice was collected and centrifuged to remove solid matter.

### DNA extraction

DNA was extracted from young leaf tissue obtained from 10 female parent plants, 10 male parent plants, and 50 plants from each of the three extreme phenotypes from the F_2_ population, as described by Murray and Thompson [24].

### Resequencing and detection of genome variations in the two parents

Paired-end (PE) and mate-pair Solexa libraries were prepared, according to the manufacturer's instructions (Illumina, Hayward, CA, USA). Shouxing and Xinguowei DNAs were randomly sheared. After electrophoresis, DNA fragments of the desired length were gel-purified. Adaptor ligation and DNA cluster preparation were performed, and the resulting DNA libraries were subjected to Solexa sequencing using an Illumina GAIIx (Illumina) platform. Low-quality reads (<20), reads with adaptor sequences, and duplicate reads were filtered out, and the remaining high-quality data were used for mapping, which was carried out with the Burrows-Wheeler alignment (BWA) software [25].

The SAMtools software [26] was used to detect single-nucleotide polymorphisms (SNPs), using the following parameters: 1) mpileup-f ref.fa-D-C 50-g-s-u; and 2) bcftools view-cegINv. The SNPs were then screened using the following criteria: 1) no less than 2× coverage depth (no less than 3× in the heterozygous locus); 2) no more than 3× the average depth (11×); and 3) discarding of all SNPs detected in repeat regions. Structural variations (SVs) were detected using Pindel 0.2.4 [27] (perl $Bin/bin/soft/pindel/bam2pindel.pl -i sort.bam -o output -s keys -om -pi 272.67) and BreakDancer [28] Max-0.0.1r61 (perl $Bin/bin/soft/breakdancer/bam2cfg.pl sort.bam -s 40 -q 35 > $breakdancer_dir/$sample.config). To obtain reliable SVs, the detected SVs were returned to the PE alignments between the parental lines and the reference, and were validated using the following criteria: 1) 2× to 100× coverage depth; and 2) a score >20 for the quality of the SV. In our results, the types of SV included insertion (INS) and deletion (DEL) (collectively, indels), interchromosomal translocation (CTX), deletion including insertion (DII), intrachromosomal translocation (ITX), and inversion (INV) [29]. The two parents were homozygous to the different alleles.

The localization of SNPs, indels, and SVs was based on annotations of gene models provided by reference genome databases (https://melonomics.net) [18], and was carried out using the snpEff software (http://snpeff.sourceforge.net/SnpEff_manual.html). The three types of polymorphism were annotated as genic (in gene regions) or intergenic (in non-gene regions). Genic SNPs, indels, and SVs were classified as exonic or intronic according to their localization. The GeneWise software [30] was used to separate the exonic SNPs into synonymous or non-synonymous types.

### Annotation of differential gene function between Shouxing and Xinguowei

We compared the non-synonymous SNPs of the two parental lines and the SVs (including indels) in exons against the Kyoto Encyclopedia of Genes and Genomes (KEGG) databases, using the BLAST program with a cutoff E-value of 1×10^−5^.

### Super-BSA pools, library construction, and high-throughput sequencing

Five different DNA pools were prepared by mixing equal amounts of DNA from the female Shouxing bulk ‘M’ (sweet) and male Xinguowei bulk ‘P’ (sour). Fifty plants were used from each of the three extreme phenotypes from the F_2_ population bulk (sweet, sour, and non-sweet non-sour). The library was constructed as described by Sun et al. [23], with small modifications. The GC content, repeated sequences, and genetic characteristics of the DNA pools were analyzed using SLAFPredict (Biomarker, Beijing, China). The genomic DNA pools were digested using the *Xho*I and *Mse*I restriction enzymes, followed by PCR amplification, fragment amplification, fragment selection, fragment extraction and amplification, and fragment sequencing using the Illumina GAIIx system.

### SLAF marker development and polymorphism analysis

All SLAF pair-end reads with clear index information were clustered based on sequence similarity, as detected by BLAT v.34 [31] (-tileSize = 11, -stepSize = 11, -minScore = 30). Sequences with over 90% identity were grouped in one SLAF locus, as described by Sun et al. [23]. Melon is a diploid species, and one locus contains at most four SLAF tags; therefore, groups containing more than four tags were filtered out as repetitive SLAFs. In this study, SLAFs with a sequence depth <164 were defined as low-depth SLAFs and were filtered out. SLAFs with 2, 3, or 4 tags were identified as polymorphic SLAFs and considered to be potential markers.

### Fine mapping of the sweet and sour traits

All markers were identified based on the parental origin of alleles M and P, according to the sequencing depth. *M*_sweet_ represented the depth for the sweet phenotype from the female line; *P*_non-sweet non-sour_ represented the depth for the non-sweet non-sour phenotype from the male line; *M*_non-sweet non-sour_ represented the depth for the non-sweet non-sour phenotype from the female parent; and *P*_sour_ represented the depth for the sour phenotype from the male line. The following ratios were calculated: *Ratio_sweet* = *M*_sweet_/*P*_non-sweet non-sour_; and *Ratio_sour* = *P*_sour_/*M*_non-sweet non-sour_. In the case that *P*_non-sweet non-sour_ = 0, *Ratio_sweet* was set to 1000; in the case that *M*_non-sweet non-sour_ = 0, *Ratio_sour* was set to 1000. The thresholds for association were set at a ratio ≥3. Although the ratio measurement employed in this analysis has not been widely used in previous studies, it was recently described to be a good approach to BSA analysis [32]. It can be inferred that a ratio ≥3 means an SNP-index ratio ≥0.5. The SNP-index indicates the proportion of reads harboring a SNP that is different from the reference sequence [33].

We performed fine mapping of the genomic region according to the sequences of the sweet and sour trait-related SLAF markers in the scaffold. We used BLAT v.34 [31] (-tileSize = 11, -stepSize = 11, -minScore = 30) for every SLAF marker (https://melonomics.net) to determine the SLAF position in the genetic map.

### Combined analysis of regions associated with sweet and sour traits and parental resequencing data

The genes in regions associated with sweet and sour traits and the differential SLAF markers were matched with the parental resequencing data using BLAT v.34 [31] (-tileSize = 11, -stepSize = 11, -minScore = 30). We used the annotation in ‘gff’ files of the genome to determine whether the markers covered the gene. We determined the variation in traits by analyzing the SNPs between genes and genome where correlated markers were located; or by analyzing the SNPs between samples (whether the SNP variation induced genetic variation and influenced the variation in the traits). Finally, we extracted the corresponding genes according to the physical location of the markers (in genes) and that correlated with the sweet and sour traits through gene function annotation.

## Results

### Variability in fruit TSS and pH in the two parents and the F_1_ and F_2_ populations

To estimate the variation in the TSS and pH of the ripe fruits, TSS and pH were measured in the parents and the F_1_ (Fengwei melon) and three F_2_ populations grown in Turpan during two growing seasons (open field in spring and autumn 2012, and greenhouse in autumn 2012). Results showed that there was significant variability in the TSS and pH traits between the parental, F_1_, and F_2_ populations (p≤0.01; Welch’s ANOVA test; Table 1). Fruits grown in the spring showed higher levels of TSS and pH than those grown in autumn. In addition, fruits grown in autumn had higher levels of TSS and pH when grown in a greenhouse than when grown in an open field. We used the greenhouse results from autumn 2012 in subsequent parent resequencing and super-BSA. Melon fruits with TSS ≥8 and pH >5.3 were considered sweet, while those with TSS <8 and pH ≤5.3 were considered sour. We characterized Shouxing as sweet (TSS >12, pH >6), Xinguowei as sour (TSS <8, pH <5), and F_1_ Fengwei as sweet and sour (TSS >12, pH <5). The F_2_ population produced four types of trait: sweet (TSS ≥8 and pH >5.3), sour (TSS <8 and pH ≤5.3), sweet and sour (TSS >12, pH <5) and non-sweet non-sour (TSS <8, pH >5.3). We selected the sweet (TSS >12, pH >6), sour (TSS <8, pH <5) and non-sweet non-sour (TSS <8, pH >5.3) pools from the F_2_ lines for subsequent super-BSA.

**Table 1:** TSS and pH data of the parental lines and F_1_ and F_2_ populations.

	Planting	Shouxing	Xinguowei	F_1_	F_2_	F_2_	F_2_
Trait	season and location	(mean ± SD)	(mean ± SD)	(mean ± SD)	(mean ± SD)	(max ± SD)	(min ± SD)
TSS	Open field in spring	13.8 ± 1.4	7.5 ± 1.1	13.2 ± 1.2	11.2 ± 1.5	15.2 ± 1.8	7.2 ± 0.6
	Open field in autumn	12.2 ± 2.1	7.1 ± 1.3	12.2 ± 0.8	10.1 ± 1.1	13.2 ± 1.8	7.0 ± 0.5
	Greenhouse in autumn	12.7 ± 1.2	6.8 ± 0.9	12.6 ± 1.4	10.9 ± 0.9	15.2 ± 1.8	6.6 ± 0.3
pH	Open field in spring	6.3 ± 0.2	4.6 ± 0.2	5.0 ± 0.6	5.5 ± 0.6	6.6 ± 0.9	4.4 ± 0.2
	Open field in autumn	6.1 ± 0.1	4.5 ± 0.3	4.8 ± 1.1	5.4 ± 0.6	6.3 ± 0.9	4.4 ± 0.4
	Greenhouse in autumn	6.1 ± 0.2	4.4 ± 0.2	4.8 ± 0.6	5.2 ± 0.6	6.2 ± 0.9	4.3 ± 0.2

Mean and standard deviation (SD) values were calculated using JMP v7.0 software.

### Discovery of polymorphism within genic regions related to fruit flavor traits

We used a Solexa Genome Analyzer II for genomic resequencing of the Shouxing and Xinguowei parental lines. Shouxing yielded 29,882,152 101-bp short sequences while Xinguowei produced 30,603,252 short sequences. We used the BWA software to match the 24,796,210 and 25,746,516 reads in the two parental lines to the reference genome, DHL92 (Table 2).

**Table 2:** Summary of the resequencing coverage.

Sample	Total reads	Total_map (%)	Identity (%)	Depth	Coverage_ratio (%)
Shouxing	29,882,152	82.98%	98.91%	13.07	93.55%
Xinguowei	30,603,252	84.13%	98.92%	13.2	94.74%

The analyses were conducted using the Map_stat_v0.1.pl software [34]. Total map (%): number of clean reads successfully mapping to the genome/total number of clean reads. Identity (%): (total number of all clean reads mapping to the genome—number of all mismatched bases)/total number of all clean reads mapping to the genome. Coverage_ratio (%): total number of bases mapping to the genome/genome size.

**Fig 1:**
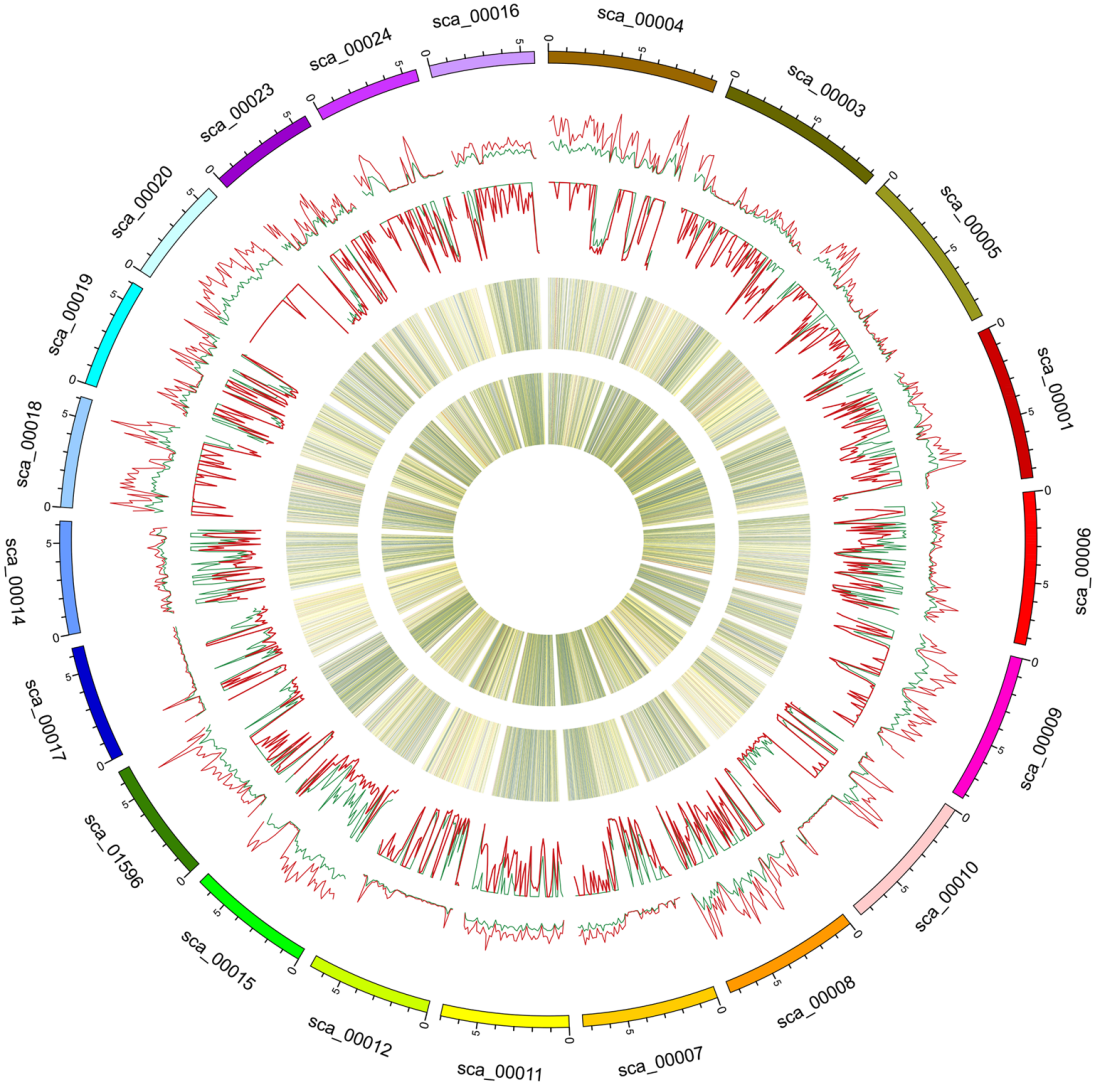
Genome sequence variations identified in Shouxing and Xinguowei using Circus programs. The first ring is the scaffold in the reference genome. The second ring is the SNP distribution in Shouxing. The third ring is the SNP distribution in Xinguowei. The fourth ring is the SV distribution in Shouxing. The fifth ring is the SV distribution in Xinguowei. The SNPs and SVs of the two parental lines are greatest on scaffold 4, i.e. 44,625 and 1,534 for Shouxing, and 42,395 and 1,560 for Xinguowei, respectively. The SNPs (8,554 and 9,612) and SVs (946 and 956) for the two respective parental lines are lowest on scaffold 21.

**Fig 2:**
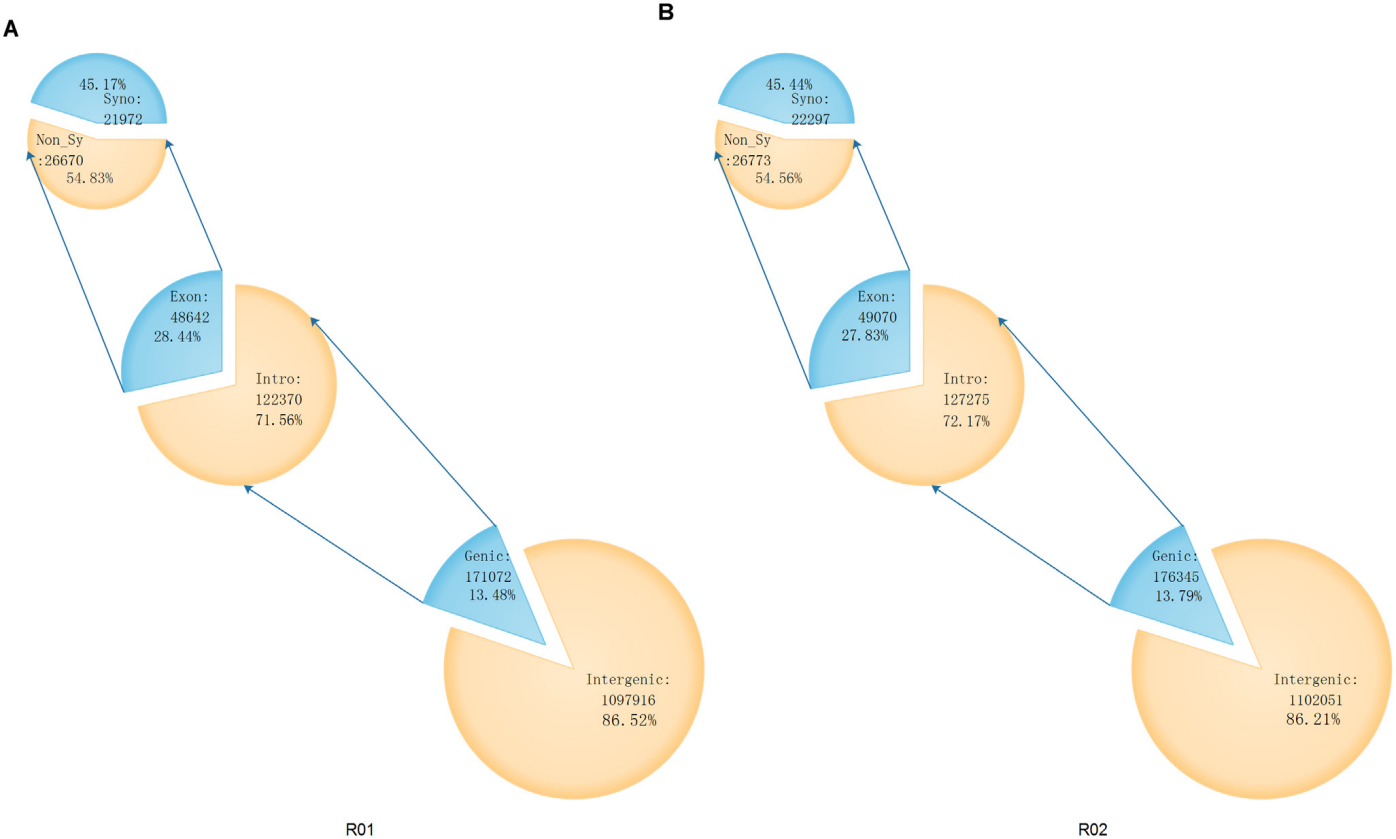
SNP annotations in Shouxing and Xinguowei. According to the annotation of the reference genome DHL92, the locations of the SNPs in the genes were classified as genic or intergenic. Genic SNPs were further classified as intronic or exonic, and SNPs in the exon region were subclassified as either synonymous or non-synonymous variations. The number and proportion of SNP polymorphisms in each class are shown. A: Shouxing; B: Xinguowei.

**Fig 3:**
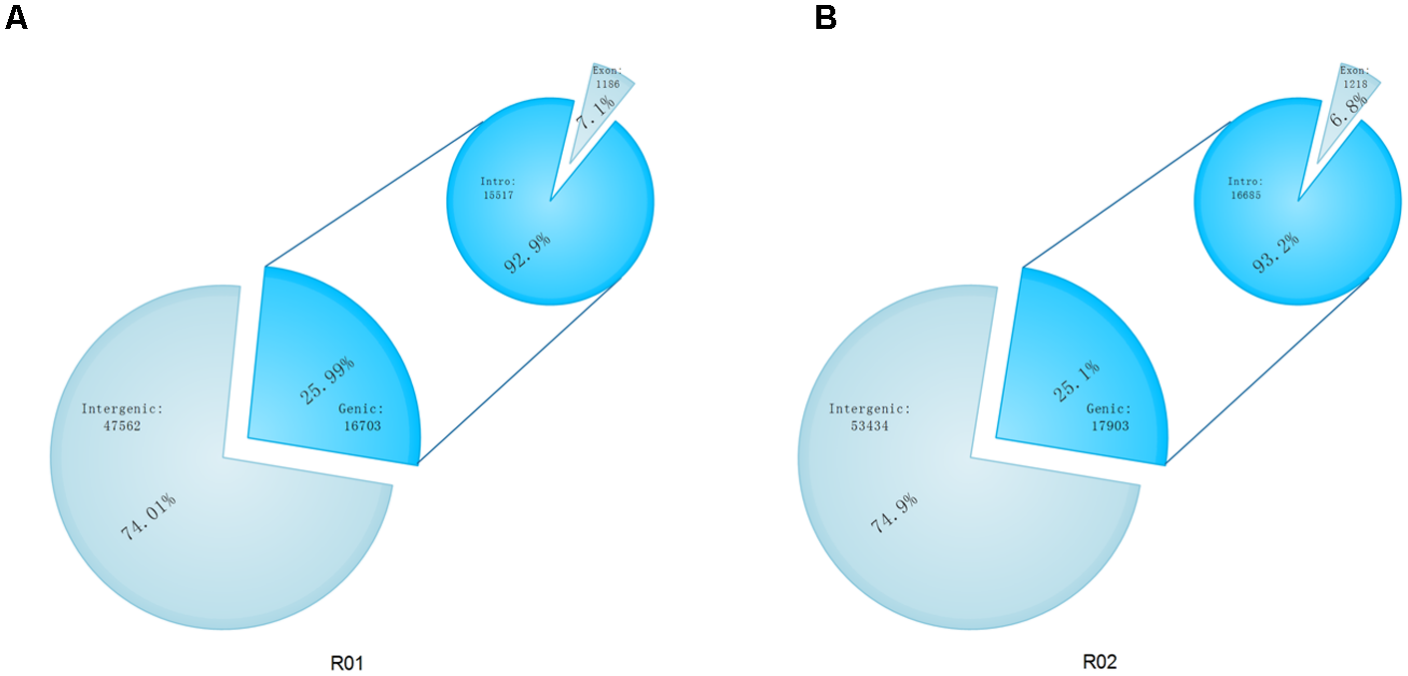
Annotation of SVs in Shouxing and Xinguowei. According to the annotation of the reference genome DHL92, the locations of the SVs in the genes were classified as genic or intergenic. Genic SNPs were further classified as intronic or exonic. The number and proportion of SV polymorphisms in each class are shown. A: Shouxing; B: Xinguowei.

The SAMtools software [26] was used to search for SNPs in the genomes of the two parental lines. There were 1,278,396 SNPs between Shouxing and DHL92, and 1,268,988 SNPs between Xinguowei and DHL92 (Fig 1). According to the differences in the nucleotide substitutions, the SNPs were either transitions (C/T or G/A, shown in red) or transversions (C/G, T/A, A/C or G/T, shown in black) (Fig 1). For both parental lines, the proportion of transitions (Ts) was higher than that of transversions (Tv). The Ts/Tv ratios for Shouxing and Xinguowei, with reference to the DHL92 genome, were 2.44 and 2.42, respectively. There were 171,072 and 176,345 genic SNPs, respectively. Of the genic SNPs in Shouxing and Xinguowei, 48,642 and 49,070 were located in exons, respectively, and included 26,670 and 26,773 non-synonymous SNPs, respectively (Fig 2A and 2B). Of the 1,152,860 differential SNPs between Shouxing and Xinguowei, 22,322 were non-synonymous. We also used the Pindel [27] and BreakDancer [28] software to detect indels and SVs in the genomes of the two parental lines (Fig 1). We identified 26,802 indels and 10,109 SVs in Shouxing melon, and 28,546 indels and 12,968 SVs in Xinguowei melon. There were 16,703 and 17,903 genic SVs (including indels), and 1,186 and 1,218 exonic SVs (including indels), in Shouxing and Xinguowei, respectively (Fig 3A and 3B). Of the 64,265 and 71,337 SVs (including indels) detected in these two parental lines, INS and DEL accounted for 94.98% and 95.54%, respectively. In Shouxing and Xinguowei, other types of SV and CTX accounted for 4.58% and 4.04%, respectively; In DELs and INS for 0.23% and 0.22%, respectively; CTX for 0.15% and 0.13%, respectively; and INV for 0.06% and 0.07%, respectively. To verify the accuracy of the detection of SNPs and SVs, we randomly selected variations in 400 SNPs and 100 SVs (INS and DEL) of 100 to 300 bp in length for validation in the two inbred lines by PCR and sequencing (S1, S2, S3 and S4 Tables). Among these, 394 SNPs and 92 SV loci could be amplified and sequenced. These data verified 98.75% of the SNPs and 92.0% of the SVs, indicating that our re-sequencing data were reliable.

### Identification of pathways associated with fruit flavor traits

Previous studies have speculated that genetic variations in melon fruit may contribute to phenotypic differences in traits [8,9,15,16]. Therefore, we focused our analysis on non-synonymous SNPs, indels, and SVs in the exons of genes in the two parental lines. We performed a preliminary examination of the genes’ functional annotations to assess the influence of genotypic variations on phenotypic variations.

Pathway enrichment analysis for the 575 Shouxing polymorphic genes in 101 KEGG pathways and the 715 Xinguowei polymorphic genes in 110 KEGG pathways enabled the identification of several pathways contributing to fruit flavor traits. The pathways identified included starch and sucrose metabolism, fructose and mannose metabolism, galactose metabolism, citrate cycle metabolism, nicotinate and nicotinamide metabolism, folate biosynthesis, and vitamin B6 metabolism (S5 Table and S6 Table). We identified four genes in Xinguowei that were annotated in the pathway for nicotinate and nicotinamide metabolism: nudix hydrolase-19, nicotinamide mononucleotide adenylyltransferase-3, nicotinate phosphoribosyltransferase, and L-aspartate oxidase. We also annotated two genes as part of the folate pathway: folylpolyglutamate synthase and bifunctional dihydrofolate reductase-thymidylate synthase. Threonine synthase, whose encoding gene was identified as polymorphic, participates in vitamin B6 metabolism, which is associated with fruit acid. The higher content of nicotinate, folate, and vitamin B6 in Xinguowei (data not shown) may be associated with variations in the genes of these pathways. In Xinguowei, three polymorphic genes were identified and annotated in the valine, leucine, and isoleucine pathways. These included: pyruvate dehydrogenase E1 component subunit beta-3, branched-chain-amino-acid aminotransferase-like protein-3, and 2-isopropylmalate synthase-2.

The three main soluble sugars in melons are sucrose, glucose and fructose. Sugar metabolism and accumulation determines the sweetness of the melon. We found polymorphisms between the two parents in the starch and sucrose pathway, fructose and mannose pathway, and galactose pathway. Organic acids in fruits are essential components that reflect fruit flavor characteristics. The citrate cycle pathway and its key steps are necessary for the accumulation of organic acids in fruits. Therefore, DNA polymorphisms in these genes could explain the differences in sweet and sour traits between the two inbred lines.

The polymorphic genes in Xinguowei annotated in the citrate cycle pathway encode pyruvate dehydrogenase, aconitate hydratase, isocitrate dehydrogenase, succinate dehydrogenase, phosphoenolpyruvate carboxykinase, succinyl-CoA ligase and 2-oxoglutarate dehydrogenase.

In both Xinguowei and Shouxing, we also identified genes encoding members of the lipoxygenase family, alcohol dehydrogenase family, and cadinene synthase, as well as genes involved in the synthesis of linalool derivatives or the nerolidol synthase of the linalool monomer.

### Super-BSA, SLAF markers and polymorphism analysis

Super-BSA analysis was carried out to genotype the bulk pools of the two parental lines and three F_2_ population: 50 individual plants with TSS ≥8 and pH >5.3, 50 with TSS <8 and pH ≤5.3, and 50 with TSS <8 and pH >5.3. The data from the above pools corroborated with each other to some extent, eliminating the need for biological replicates.

**Fig 4:**
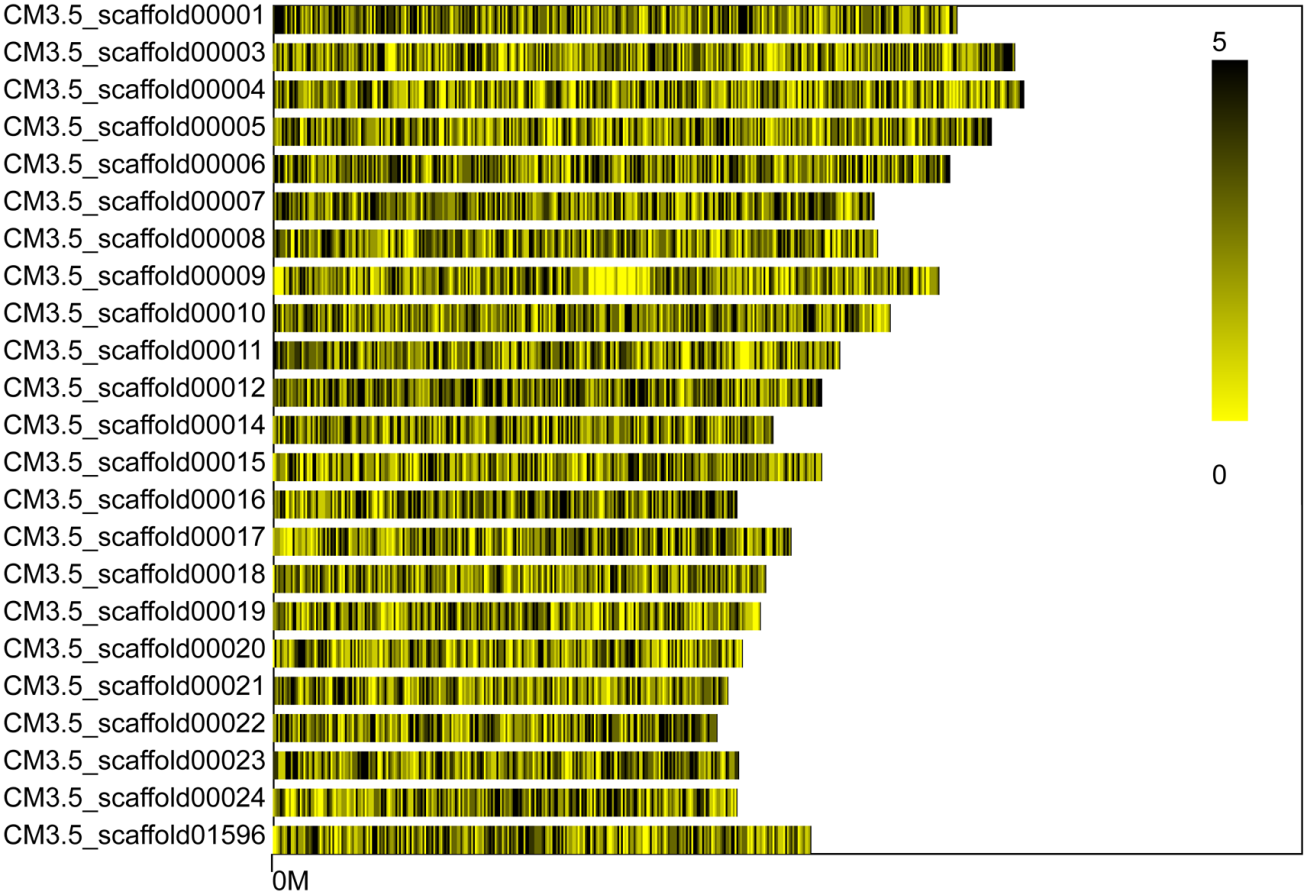
SLAF distribution on the >5 Mb scaffolds obtained using the SLAF.distribution.pl software. The reference genome used in the analysis was the scaffold that had not been spliced into chromosomes. The SLAF distributions on the scaffolds >5 Mb were selected and counted. The black vertical line on the x axis represents the position of the SLAF in the scaffold, while the y axis represents the number of scaffolds >5 Mb. Dark and light colors denote the SLAF marker numbers at these loci.

We obtained 12,438,270 reads (Table 3), and used the BLAT matching software to cluster these reads (http://www.blat.net) and map them to the reference DHL92 genome after correction. SLAFs were selected with depths larger than 10×, resulting in a total of 46,087 SLAF tags being obtained with an average depth of 161.81×. Fig 4 shows the even distribution of SLAF tags on the scaffolds (>5 Mb).

**Table 3:** Summary of SLAF-seq coverage.

Sample	Read length (bp)	Read number	GC percentage
Shouxing	80	845,612	86.67%
Xinguowei	80	1,703,803	83.65%
Sweet	80	3,113,710	85.33%
Sour	80	3,671,547	82.70%
Non-sweet, non-sour	80	3,103,598	84.47%

The analyses were conducted using fq_stat_sample software (Biomarker, Beijing, China).

**Fig 5:**
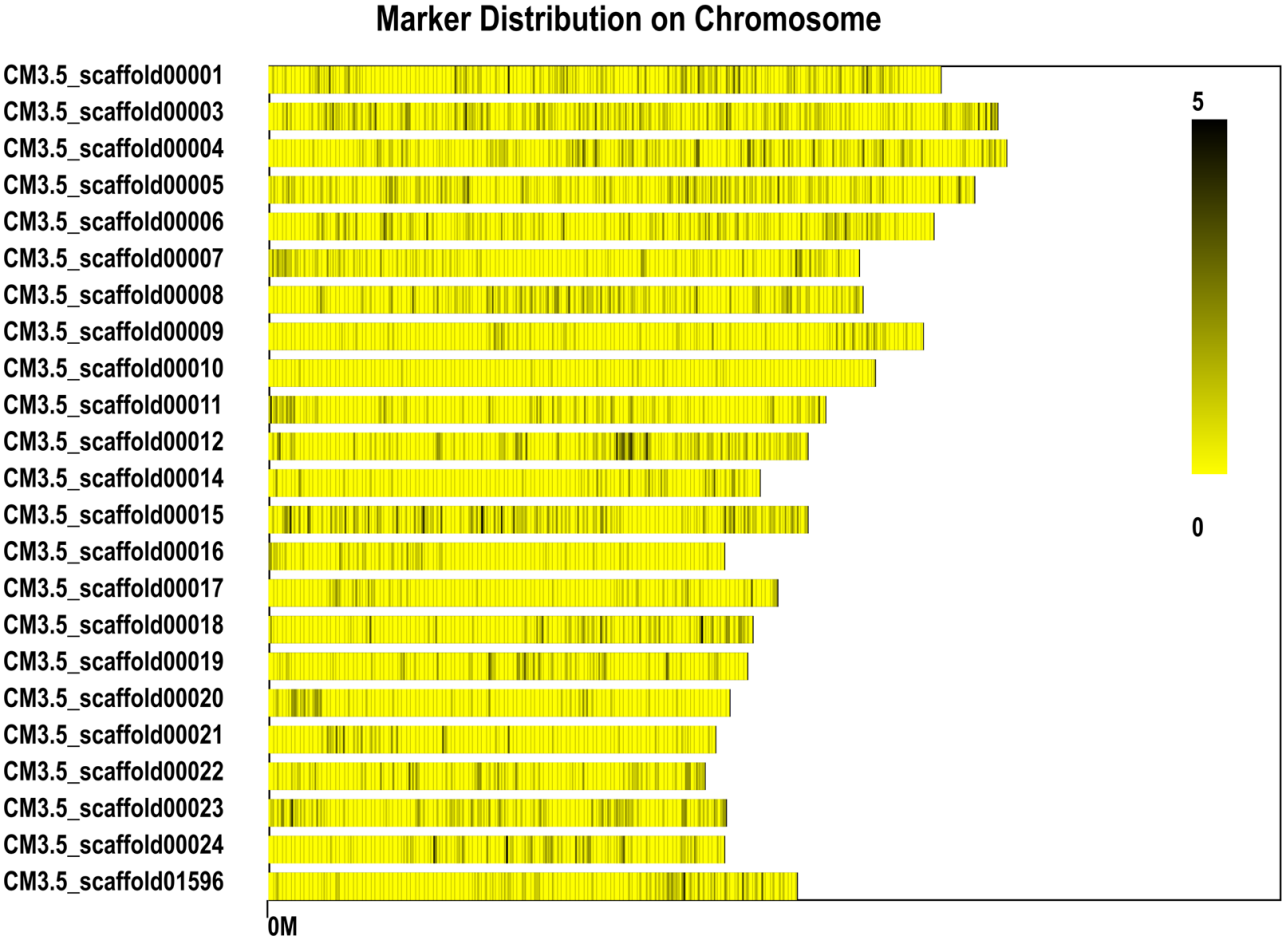
Diff_Marker distribution on the >5 Mb scaffolds assessed using the SLAF.distribution.pl software. The black vertical lines on the x axis represent the location of the marker in the scaffold. The y axis represents the scaffold number. Dark and light colors denote the quantity of SLAF markers at these loci.

We identified the polymorphic loci of 4,480 SLAF markers (Fig 5) and classified these as SNPs, enzyme locus variations, or indels (Table 4). The number of markers was greatest on CM3.5_scaffold00003 (117), and lowest on CM3.5_scaffold00010 (11) (Fig 5).

**Table 4:** Summary of the SLAF tag and polymorphic markers.

Type	SNP	EPSNP	INDEL	No polymorphism	Unknown	Repeat	Total
Number	4,326	99	55	39538	888	1181	46087
Percent	9.38%	0.21%	0.11%	85.78%	1.92%	2.56%	100%

SNP, EPSNP, and indel analyses of SLAF markers were conducted using the Group_class_ref.pl software.

### Association mapping of genes related to sweet and sour traits

We compared the 4,480 SLAF markers with the allelic frequencies of the parental lines and found 2,800 sweet trait-related SLAF markers and 2,711 sour trait-related SLAF markers from the parents.

**Fig 6:**
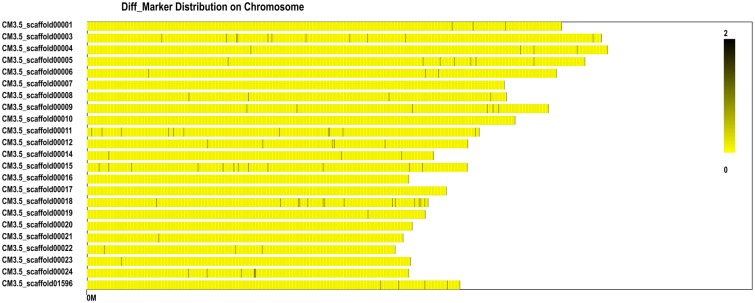
Distribution of sweet trait-related Diff_Marker on the scaffolds determined using the SLAF.distribution.pl software. The x axis refers to the location of the scaffold. The y axis represents the number of the scaffold. The black line represents the position of Diff_Marker on the scaffold.

**Fig 7:**
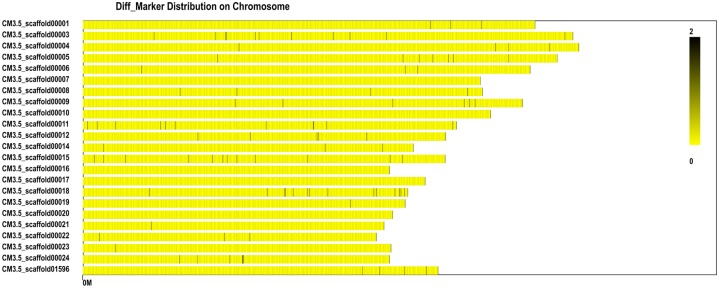
Distribution of sour trait-related Diff_Marker on the scaffolds determined using the SLAF.distribution.pl software. The x axis refers to the location of the scaffold. The y axis represents the number of the scaffold, and the black line represents the position of Diff_Marker on the scaffold.

**Fig 8:**
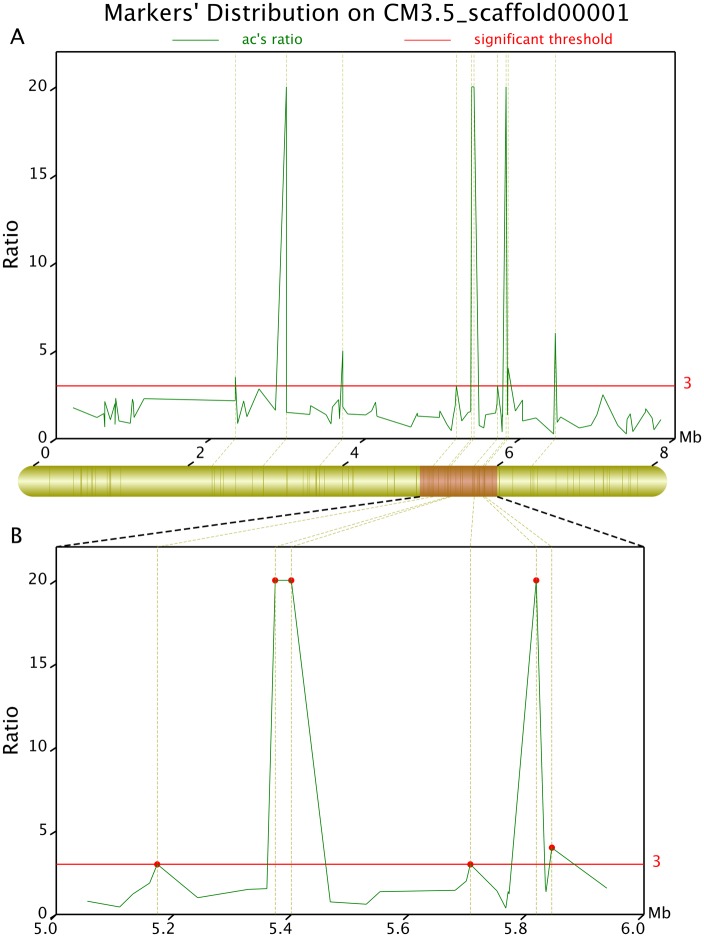
Differential ratio distribution of the sweet trait-related differential markers on CM3.5_scaffold00001 determined using the Geno_ratio.pl software. The x axis represents chromosomal position. The y axis shows the difference ratio. The upper part of the figure presents the global distribution of the differential markers, while the lower part shows the local distribution. The y axis values at the red lines suggest that the corresponding markers are intensively correlated with target traits. Higher values indicate a more intensive correlation. When *Ratio_R*°>20, the correlation intensity is 20.

**Fig 9:**
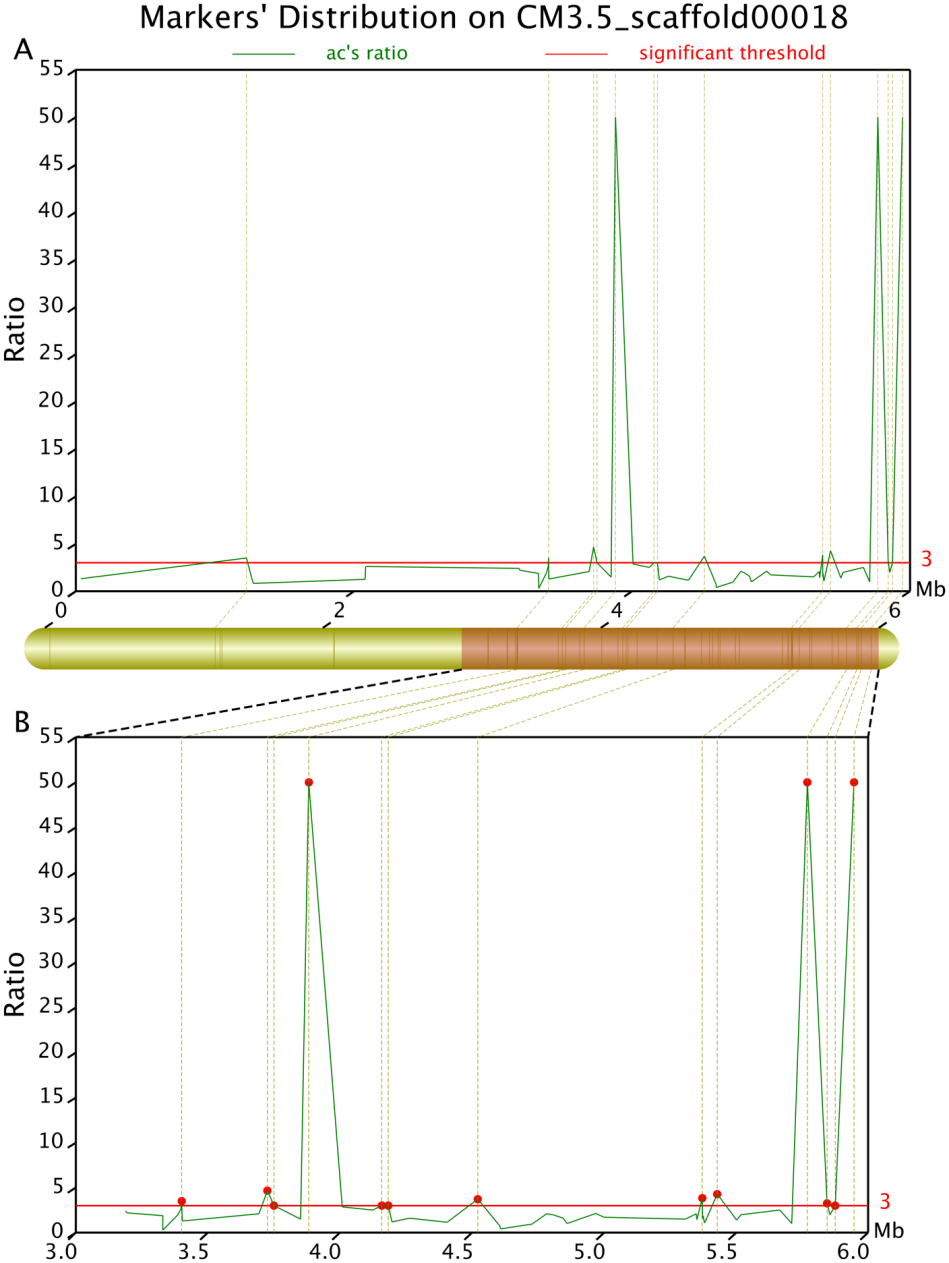
Differential ratio distribution of the sour trait-related differential markers on CM3.5_scaffold00018 determined using the Geno_ratio.pl software. The x axis represents chromosomal position. The y axis shows the difference ratio. The upper part of this figure presents the global distribution of the differential markers, while the lower part shows the local distribution. The y axis values at the red lines suggest that the corresponding markers are intensively correlated with the target traits. Higher values indicate a more intensive correlation. When *Ratio_R*°>20, the correlation intensity is 20.

We identified 114 differential SLAF markers related to the sweet trait (*Ratio_sweet* ≥3; Fig 6) and 215 related to the sour trait (*Ratio_sour* ≥3; Fig 7). Examination of the distribution of the differential SLAF markers on the CM3.5_scaffold00001 suggested that they correlated most intensively with the sweet trait, showing a coverage area of 0.9 Mb containing 10 markers (marking density of 0.09 Mb/marker) (Fig 8). The markers in the CM3.5_scaffold00018 were most intensively correlated with the sour trait, presenting a coverage area of 2.75 Mb containing 14 markers (marking density of 0.2 Mb/marker) (Fig 9).

The presence of three or more differential SLAF markers (*Ratio_sweet* ≥3) in succession helped identifying the regions associated with the sweet and sour traits. There were six sweet trait-related regions containing 13 differential SLAF markers and 62 genes (Table 5), and 23 sour trait-related regions containing 48 differential SLAF markers and 185 genes (Table 6).

**Table 5:** Association regions, SLAF markers, and number of genes related to the sweet trait.

Scaffold ID	Start	End	Size (bp)	SLAF marker	Gene number
CM3,5_scaffold0001	5.372,000	5,401,000	29,000	SLAF3367	2
				SLAF3370	
CM3,5_scaffold0004	5,312,000	5,319,000	7,000	SLAF6320	0
				SLAF6321	
CM3,5_scaffold0006	861,000	875,000	14,000	SLAF8215	2
				SLAF8216	
CM3,5_scaffold0016	1,793,000	2,126,000	333,000	SLAF18729	54
				SLAF18745	
				SLAF18775	
CM3,5_scaffold0023	2,740,000	2,845,000	105,000	SLAF24883	4
				SLAF24901	
CM3,5_scaffold0045	1,181,000	1,195,000	14,000	SLAF38671	0
				SLAF38677	

Scaffold ID: scaffold number; Start: the starting position of the candidate region; End: the end position of the candidate region; Size: the size of the region, in units of bp; SLAF marker: the differential SLAF markers in the region; Gene number: the number of genes in the region. The data were obtained using the Geno_ratio.pl software.

**Table 6:** Association regions, SLAF markers, and number of genes related to the sour trait.

Scaffold ID	Start	End	Size (bp)	SLAF marker	Gene number
CM3,5_scaffold00003	2,631,000	2,653,000	22,000	SLAF4675	0
				SLAF4679	
CM3,5_scaffold00003	3,188,000	3,256,000	68,000	SLAF4736	4
				SLAF4745	
CM3,5_scaffold00004	7,621,000	7,864,000	243,000	SLAF6659	15
				SLAF6693	
CM3,5_scaffold00009	7,143,000	7,243,000	100,000	SLAF12456	2
				SLAF12467	
CM3,5_scaffold00011	4,255,000	4,504,000	249,000	SLAF14408	33
				SLAF14409	
				SLAF14440	
CM3,5_scaffold00018	3,724,000	3,750,000	26,000	SLAF20908	2
				SLAF20910	
CM3,5_scaffold00018	4,157,000	4,183,000	26,000	SLAF20972	1
				SLAF20979	
CM3,5_scaffold00018	5,769,000	5,845,000	76,000	SLAF21206	8
				SLAF21215	
CM3,5_scaffold00018	5,874,000	5,947,000	73,000	SLAF21222	4
				SLAF21231	
CM3,5_scaffold00024	2,947,000	2,955,000	8,000	SLAF25822	1
				SLAF25825	
CM3,5_scaffold00032	4,022,000	4,025,000	3,000	SLAF31553	0
				SLAF31554	
CM3,5_scaffold00040	2,861,000	2,988,000	127,000	SLAF36320	6
				SLAF36334	
CM3,5_scaffold00054	362,000	438,000	76,000	SLAF42241	2
				SLAF42254	
CM3,5_scaffold00054	833,000	987,000	154,000	SLAF42301	7
				SLAF42321	
CM3,5_scaffold00054	1,441,000	1,480,000	39,000	SLAF42384	0
				SLAF42394	
CM3,5_scaffold00054	1,920,000	2,240,000	320,000	SLAF42476	29
				SLAF42498	
				SLAF42509	
CM3,5_scaffold00054	2,287,000	2,313,000	26,000	SLAF42523	0
				SLAF42526	
CM3,5_scaffold00055	2,323,000	2,397,000	74,000	SLAF42926	5
				SLAF42936	
CM3,5_scaffold00083	196,000	261,000	65,000	SLAF50072	7
				SLAF50085	
CM3,5_scaffold00088	141,000	253,000	112,000	SLAF50944	5
				SLAF50974	
CM3,5_scaffold00089	595,000	867,000	272,000	SLAF51212	42
				SLAF51247	
CM3,5_scaffold00095	491,000	496,000	5,000	SLAF52032	0
				SLAF52035	
CM3,5_scaffold00114	53,000	120,000	67,000	SLAF53315	12
				SLAF53325	

Scaffold ID: scaffold number; Start: the starting position of the candidate region; End: the end position of the candidate region; Size: the size of the region, in units of bp; SLAF marker: the differential SLAF markers in the region; Gene number: the number of genes in the region. The data were obtained using the Geno_ratio.pl software.

**Fig 10:**
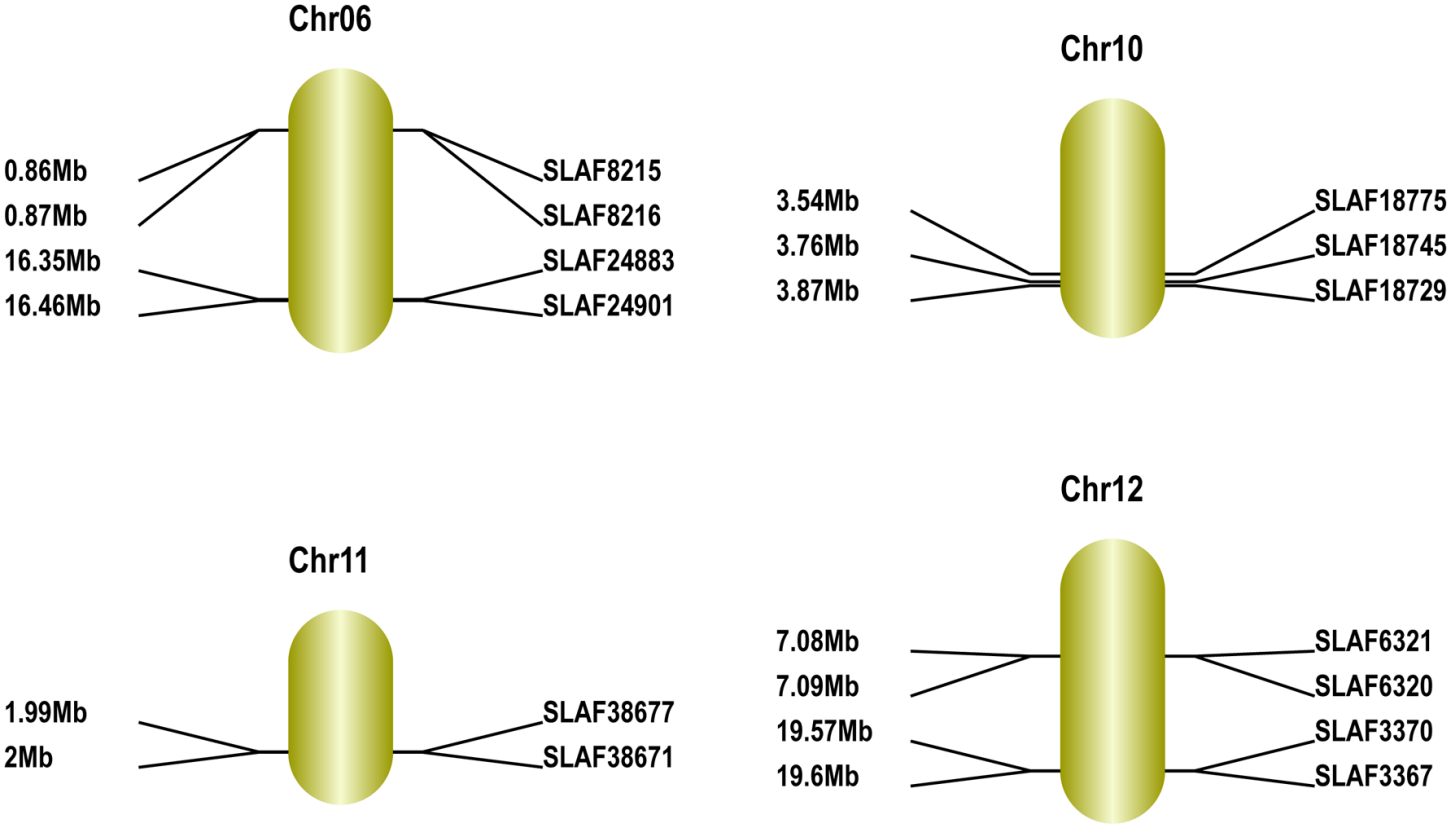
Maps of sweet trait-related genomic regions in melon obtained using the GenticMapDrawer.pl l software. Chromosome numbers are according to Garcia-Mas et al. [18]. Distances from the top of each chromosome are marked on the left side, and marker names are on the right side of each linkage group.

**Fig 11:**
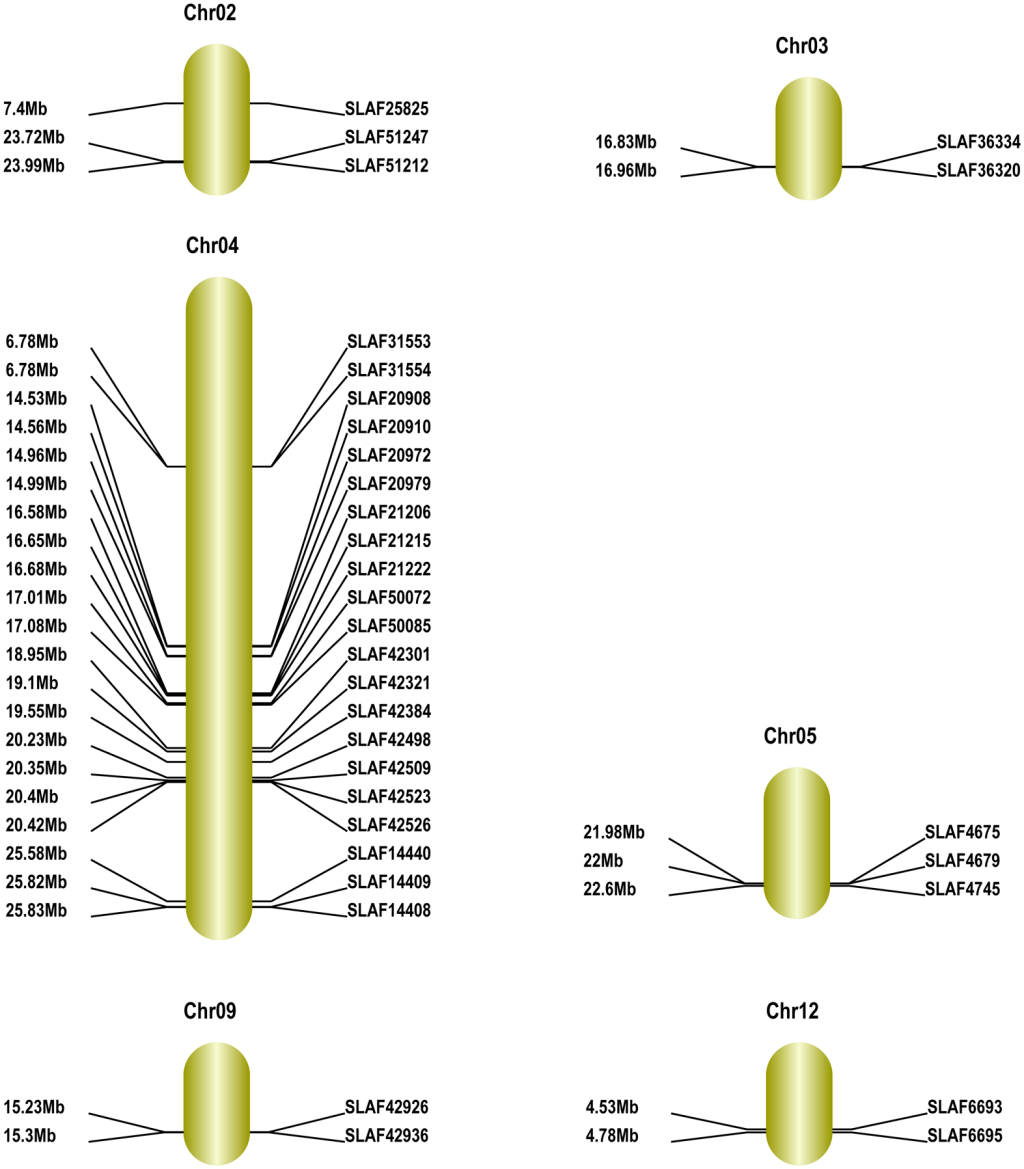
Maps of sour trait-related genomic regions in melon obtained using the GenticMapDrawer.pl l software. Chromosome numbers are according to Garcia-Mas et al. [18]. Distances from the top of each chromosome are marked on the left side, and marker names are on the right side of each linkage group.

To anchor the above 13 sweet trait-related and 48 sour trait-related SLAF markers in the scaffolds to the genetic map, we performed BLAT analysis of every SLAF marker in the scaffolds against the genetic map (https://melonomics.net/genetic_map/map_set_info). We mapped the 13 sweet trait-related SLAF markers to between 0.86 and 26.46 kb on chromosome 6; 3.54 and 3.87 kb on chromosome 10; 1.99 and 2.01 kb on chromosome 11; and 7.09 and 19.60 kb on chromosome 12 (Fig 10 and Table 7). We also mapped 38 of the 48 sour trait-related SLAF markers to between 7.4 and 23.73 kb on chromosome 2; 16.84 and 16.96 kb on chromosome 3; 6.79 and 25.84 kb on chromosome 4; 21.98 and 22.6 on chromosome 5; 15.23 and 15.3 kb on chromosome 9; and 4.54 and 4.78 kb on chromosome 12 (Fig 11 and Table 8). We discarded 10 sour trait-related SLAF markers, each of which mapped to more than two different locations in the genome. We used the method described by Harel-Beja et al. [15] to measure the glucose and sucrose content (i.e. sweetness) of the melons, and the method of Cohen et al. [8] to measure the pH, citrate content and malic acid content (i.e. acidity). Based on the genetic mapping positions, we mapped sucrose QTLs, TSS QTLs and glucose QTLs to linkage groups LG2, LG3, LG4, LG5 and LG8 [14], while pH, citrate and malate QTLs were mapped to LG4, LG8, LG11 and LG12 [7,9,14,15].

**Table 7:** Fine maps of sweet trait-related genomic regions in melon.

Chr ID	SLAF_Marker	Start	End
chr06	SLAF8215	861471	861868
	SLAF8216	874069	874414
	SLAF24883	16359185	16359588
	SLAF24901	16462572	16462949
chr10	SLAF18729	3871880	3872260
	SLAF18745	3766227	3766593
	SLAF18775	3540166	3540533
chr11	SLAF38671	2006296	2006657
	SLAF38677	1993550	1993930
chr12	SLAF3367	19603319	19603727
	SLAF3370	19575933	19576314
	SLAF6320	7090425	7090799
	SLAF6321	7085133	7085501

Fine maps obtained using Geno_ratio.pl software.

**Table 8:** Fine maps of sour trait-related genomic regions in melon.

Chr ID	SLAF_Marker	Start (bp)	End (bp)
chr02	SLAF25825	7403095	7403459
	SLAF51212	23995375	23995750
	SLAF51247	23724902	23725285
chr03	SLAF36320	16960230	16960636
	SLAF36334	16835196	16835556
chr04	SLAF14408	25831909	25832303
	SLAF14409	25820933	25821305
	SLAF14440	25583901	25584279
	SLAF20908	14536504	14536903
	SLAF20910	14561186	14561575
	SLAF20972	14969782	14970159
	SLAF20979	14994484	14994835
	SLAF21206	16581184	16581579
	SLAF21215	16655883	16656291
	SLAF21222	16686457	16686842
	SLAF31553	6787495	6787875
	SLAF31554	6785956	6786336
	SLAF42301	18951021	18951406
	SLAF42321	19102836	19103188
	SLAF42384	19558988	19559337
	SLAF42498	20239883	20240269
	SLAF42509	20356232	20356621
	SLAF42523	20404231	20404628
	SLAF42526	20428858	20429223
	SLAF50072	17017737	17018086
	SLAF50085	17081442	17081835
chr05	SLAF4675	21980847	21981223
	SLAF4679	22001441	22001854
	SLAF4745	22604484	22604854
chr09	SLAF42926	15231134	15231547
	SLAF42936	15304483	15304845
chr12	SLAF6659	4781508	4781871
	SLAF6693	4539737	4540115

Fine maps obtained using Geno_ratio.pl software.

### Combined analysis of regions associated with sweet and sour traits and parental resequencing data

We studied the genes from the regions associated with sweet and sour traits, and found that some of the SLAF differential markers (13 sweet trait-related and 48 sour trait-related) matched the genome-wide genetic variation in the parental resequencing data. Some sweet (62) and sour (185) trait-related variations within the associated regions showed polymorphisms between the two parents, including SNPs (70, 247) and SVs (38, 106) (Table 9). Comparative analysis of SLAF markers and parental resequencing data showed that only one marker (SLAF18745), located in one gene, N-acetylglucosaminyl transferase III (MELO3C011944T1), was correlated with the sweet trait. However, seven SLAF markers located in eight genes were associated with the sour trait; these were: four uncharacterized genes (MELO3C009704T1, MELO3C009705T1, MELO3C022688T1, MELO3C026894T2); *Mus musculus* squamous cell carcinoma antigen 2 (Scca2) (MELO3C020002T1); cadmium-induced protein AS8-like, transcript variant 2 (MELO3C022756T1); receptor-like protein kinase-like (MELO3C025794T1); and NAC transcription factor 29-like (MELO3C026251T1). These SLAF markers for sweet and sour traits mapped to the parental resequencing data (Table 10 and S7 Table). Therefore, there was rich variation in sweet and sour trait-related genes within the two parental lines compared with DHL92.

**Table 9:** The polymorphic types of sweet and sour trait-related genes mapped to the parental genome.

Sample	Sweet			Sour		
	Total genes	SNP	SV	Total genes	SNP	SV
Shouxing	62	20	16	185	130	58
Xinguowei	62	50	22	185	117	48

Polymorphic type mapping to the parental genome was performed using stat_table.pl software.

**Table 10:** Sweet and sour trait-related genes mapped to the parental genome using Gene_Func_Anno_Pipline.pl software.

SLAF marker	Gene ID	Functional genes	Associated traits
SLAF18745	MELO3C011944T1	N-acetylglucosaminyltransferase III	Sweet
SLAF14409	MELO3C009704T1 MELO3C009705T1	Uncharacterized	Sour
SLAF36334	MELO3C020002T1	*Mus musculus* squamous cell carcinoma antigen 2 (Scca2)	Sour
SLAF42321	MELO3C022688T1	Uncharacterized	Sour
SLAF42509	MELO3C022756T1	Cadmium-induced protein AS8-like, transcript variant 2	Sour
SLAF50072	MELO3C025794T1	Receptor-like protein kinase-like	Sour
SLAF51212	MELO3C026251T1	NAC transcription factor 29-like	Sour
SLAF53325	MELO3C026894T2	Uncharacterized	Sour

## Discussion

The rapid development of sequencing technologies and bioinformatics tools has provided a starting point for unraveling the tremendous genetic diversity that occurs in the genome. Genetic variations on a genome-wide scale has been reported in several model organisms [17,29,35–37]. Techniques such as parental genome resequencing and BSA have helped identifying markers linked to genes or QTLs. These markers can be further used in the construction of genetic maps and in high-throughput genotyping polymorphisms (SSRs, SVs, or indels). We resequenced the two parental lines (Shouxing and Xinguowei) of the Fengwei melon to uncover nearly two million SNPs, indels, and SVs, and identified 1290 polymorphic genes associated with sweet and sour traits. Some of these genes are involved in metabolic pathways for sucrose, organic acids, aromas, and vitamins. Our study provides the first report on genome-wide patterns of genetic variation in melon, and will be valuable to future genotype-phenotype studies and molecular breeding.

Melon fruits accumulate various levels of soluble sugars, organic acids, vitamins, and aromatic volatiles [5], and these compounds affect fruit quality through complex networks of metabolic pathways that are active during fruit ripening. Transcriptome and gene cloning have identified the metabolic pathways for sugars that are related to sucrose accumulation in melon fruit [5]. Our results demonstrated that the two parents of Fengwei melon (Xinguowei and Shuoxing) showed polymorphisms of genes in several pathways involved in the biosynthesis of sucrose, fructose, mannose, and galactose. DNA polymorphisms in genes coding for components of these pathways may account for the differences in sweet taste between the two parents. Moreover, a transcriptome study that we have carried out has identified differential expressions of these pathways and genes in melon fruit (unpublished data).

Studies of organic acids in other fruit species have shown that the acid content of a fruit is determined by the balance between acid synthesis and degradation. The genes associated with the citrate cycle pathways have been documented to play a critical role in the accumulation of organic acids in fruits [38–44]. Our current findings reveal that the two parents showed polymorphisms of genes related to the citrate cycle pathways. DNA polymorphisms in genes involved in these pathways may contribute to the differences in sour taste between the two parents. Indeed, our transcriptome study showed differing expressions of the genes involved in the citrate cycle pathways in melon fruit (unpublished data). Recently, a pH-trait gene (CmPH) that has a major effect on fruit acidity has been cloned by map-based techniques and characterized in melon [45]. In future studies, the mechanisms of organic acid accumulation in melon fruit could be further explored by the continuous development of sour traits in melon fruit, and research into comparative genomics and transcription in fruits of other species.

The lipoxygenase family of genes governs fruit aroma, which is an important trait contributing to fruit quality [46]. The release of aromatic substances signifies the maturity of the melon fruit. Aromatic substances are closely associated with the health and nutrition of humans and are indices for evaluating melon fruit quality. We identified genes annotated as encoding members of the lipoxygenase family in the two parental lines, including genes that play roles in the ripening and aging processes in melon fruit such as ethylene synthesis, after-ripening, softening, and aroma formation [47,48]. We also identified genes encoding the alcohol dehydrogenase family that participates in ethylene signal transduction and the biosynthesis of aromatic substances [49–51], cadinene synthase that catalyzes farnesyl pyrophosphate cyclization to form (+)-δ-cadinene [52], and genes involved in the synthesis of linalool derivatives or the nerolidol synthase of the linalool monomer [53]. We also identified polymorphic genes in Xinguowei that were annotated in the metabolic pathways for valine, leucine, isoleucine, nicotinate, and nicotinamide. Valine, leucine, and isoleucine are precursors involved in the synthesis of most branched ester aromatic substances [54,55]. DNA polymorphisms in genes associated with the above pathways may also contribute to differences in taste between the two parents. Our transcriptome study also demonstrated differing expressions of these pathways in melon fruit (unpublished data).

Sherman et al. [56] combined BSA and microarrays to map the pH trait to chromosome 8 in melon. Although Harel-Beja et al. [15] measured the sweetness in melons with respect to glucose and sucrose content, none of their experiments overlapped with ours. We mapped pH and acidity (i.e. sourness) traits to chromosome 12 (LG12), as also reported in a previous study by Cohen et al. [8]. We attribute the differences in the mapping data between our work and previous studies to the different accessions used. Compared with ‘Dulce’ and ‘PI414723’, the Hami melon inbred lines Shouxing and Guowei (sour-taste Hami melon lines bred from high-dose Co^60^ γ-ray irradiation) may have different genes related to sweet and sour traits. Some of the crucial fruit quality traits such as flavor, aroma, and vitamin content, have not received enough attention. The polymorphic genes annotated in our study provide abundant resources for research concerning the molecular mechanisms associated with melon fruit traits.

Takagi et al. [57] used BSA in combination with high-throughput sequencing technologies, including QTL-seq and BSR-seq, to fine-map partial resistance to fungal rice blast disease and seedling vigor trait in rice. Trick et al. [58] used BSA and high-throughput sequencing to map *Gpc-B1*, a wheat quantitative trait locus associated with an increased grain content of protein, zinc, iron, and gl3, while Liu et al. [59] mapped the phenotypic mutants of maize using these techniques. We combined SLAF-seq and super-BSA to identify rapidly the genomic regions associated with sweet and sour traits, using members of the F_2_ population with extreme phenotypes.

The success of fine mapping the sweet and sour traits using the super-BSA technology provides strong technical support for its future application. In this method, high-density scanning of 10,000+ SNPs on large-scale bulk pools generated a massive amount of sequencing information that covered the whole genome. We compared the difference in the frequency of occurrence of different genes marked by a SNP in the two bulk pools and determined the molecular markers and fine mapping regions closely correlated with the traits. Thus, we directly used the sequencing results for molecular marker development. Moreover, our research provides a convenient way to develop trait-related functional molecular markers for molecular marker-assisted breeding.

Methods combining the efficiency of genome-wide variation mining and genomic mapping, including resequencing, super-BSA, QTL-seq [57], and BSR-seq [58,59], will dramatically accelerate crop improvement in a cost-effective manner. The technologies that take full advantage of the rapidly declining cost of genome sequencing are expected to contribute to the on-going efforts aimed at addressing the world food security problem by reducing breeding time.

## Supporting Information

S1 Table 200 SNP loci chosen from Shouxing for validation by PCR and sequencing. (XLSX)

S2 Table 200 SNP loci chosen from Xinguowei for validation by PCR and sequencing. (XLSX)

S3 Table 50 SV loci chosen from Shouxing for validation by PCR and sequencing. (XLSX)

S4 Table 50 SV loci chosen from Xinguowei for validation by PCR and sequencing. (XLSX)

S5 Table Statistical analysis of KEGG pathway gene variations between Shouxing and Xinguowei determined using the Gene_Func_Anno_Pipline.pl software. (XLS)

S6 Table Variations in genes for flavor, aroma, and vitamins between Shouxing and Xinguowei determined using the Gene_Func_Anno_Pipline.pl software. (XLS)

S7 Table Mapping of genes on parental chromosomes related to sweet and sour traits. (XLS)
